# Sevoflurane Induces Neurotoxicity in the Animal Model with Alzheimer’s Disease Neuropathology via Modulating Glutamate Transporter and Neuronal Apoptosis

**DOI:** 10.3390/ijms23116250

**Published:** 2022-06-02

**Authors:** Chunxia Huang, John Man Tak Chu, Yan Liu, Vivian Suk Wai Kwong, Raymond Chuen Chung Chang, Gordon Tin Chun Wong

**Affiliations:** 1 Department of Anaesthesiology, School of Clinical Medicine, LKS Faculty of Medicine, The University of Hong Kong, Hong Kong SAR, China; huangchunxiayun@sina.cn (C.H.); jmtchu@hku.hk (J.M.T.C.); lake314159@163.com (Y.L.); kwongsw@hku.hk (V.S.W.K.); 2 Laboratory of Neurodegenerative Diseases, School of Biomedical Sciences, LKS Faculty of Medicine, The University of Hong Kong, Hong Kong SAR, China; 3 State Key Laboratory of Brain and Cognitive Sciences, The University of Hong Kong, Hong Kong SAR, China

**Keywords:** sevoflurane, cognitive function, 3 × Tg mice, glutamate transporter, MAPK, apoptosis

## Abstract

Perioperative neurocognitive disorders are frequently observed in postoperative patients and previous reports have shown that pre-existing mild cognitive impairment with accumulated neuropathology may be a risk factor. Sevoflurane is a general anesthetic agent which is commonly used in clinical practice. However, the effects of sevoflurane in postoperative subjects are still controversial, as both neurotoxic or neuroprotective effects were reported. The purpose of this study is to investigate the effects of sevoflurane in 3 × Tg mice, a specific animal model with pre-existing Alzheimer’s disease neuropathology. 3 × Tg mice and wild-type mice were exposed to 2 h of sevoflurane respectively. Cognitive function, glutamate transporter expression, MAPK kinase pathways, and neuronal apoptosis were accessed on day 7 post-exposure. Our findings indicate that sevoflurane-induced cognitive deterioration in 3 × Tg mice, which was accompanied with the modulation of glutamate transporter, MAPK signaling, and neuronal apoptosis in the cortical and hippocampal regions. Meanwhile, no significant impact was observed in wild-type mice. Our results demonstrated that prolonged inhaled sevoflurane results in the exacerbation of neuronal and cognitive dysfunction which depends on the neuropathology background.

## 1. Introduction

A proportion of postoperative patients may experience acute or even prolonged cognitive decline, a phenomenon known as perioperative neurocognitive disorders [1]. It is characterized by the impairment of different cognitive domains and neuroinflammation has been associated with pathogenesis [1]. Sevoflurane (2,2,2-trifluoro-1-(trifluoromethyl) ethyl fluoromethyl ether) is a common volatile general anesthetic agent used in most of the operations. In addition to the effects on consciousness and pain, sevoflurane demonstrates distinct favorable pharmacological features [2]. In the central nervous system (CNS), sevoflurane was shown to have neuroprotective effects in different brain disorders. For cerebral ischemia/reperfusion, sevoflurane exposure attenuates brain damage in Sprague Dawley rats through inhibiting autophagy and apoptosis [3]. It also alleviates liposaccharide-induced neuroinflammation, neuronal cell loss, and cognitive deficits [4]. For hemorrhagic shock, sevoflurane postconditioning exerts neuroprotection by improving mitochondrial health in the hippocampus and enhancing spatial learning and memory function [5].

Nevertheless, apart from the beneficial effects, contradictory results were also observed in other conditions that sevoflurane is shown to be neurotoxic. In pregnant rats, fetal exposure of sevoflurane results in neurocognitive dysfunction of the offspring [6]. Molecular pathways involved in this neurotoxicity include enhancing inflammatory cytokines and inhibiting growth factors such as brain-derived neurotrophic factor and nerve growth factor in the hippocampus [6]. The neurotoxicity of sevoflurane is also observed in the animal model with type 2 diabetes, whereby cognitive function is severely impaired in the diabetic rat after sevoflurane exposure and chronic hippocampal inflammation is involved [7]. These discrepant findings indicate the differential role of sevoflurane that can be either neuroprotective or neurotoxic. The final outcome may vary which depends on the development stage of neurons and the pathological background [8]. Thus, it is important to understand when sevoflurane is neuroprotective or neurotoxic, as well as the underlying mechanisms behind these effects. From clinical reports, the patients diagnosed with pre-existing cognitive impairment are at higher risk of developing perioperative neurocognitive disorder [9,10]. For those patients, neuropathology deposit such as tau and Aβ aggregates are frequently observed [11]. It leads to the possibility that general anesthetics may have detrimental effects in the subject with accumulated neuropathology. It is therefore of interest to investigate the potential neurotoxicity of sevoflurane in the brain with pre-existing neuropathology and the mechanisms involved.

Previous studies have shown that sevoflurane could activate the excitatory glutamate neurotransmission [12] through triggering the imbalance of neuronal inhibitory/excitatory activities [13]. In other cognitive disorders such as Alzheimer’s disease (AD), excessive glutamate accumulation causes overactivation of NMDA receptors and triggers calcium influx [14]. Increased intracellular calcium ion concentration induces the calcium signaling cascade and further activates the MAP kinase (MAPK) cascade, which mediates downstream apoptotic signals in central nervous system [15,16]. In view of these findings, we speculate that sevoflurane may exaggerate excitatory glutamate neurotransmission in the subjects with neuropathology background, with the possibility of exacerbating excitotoxicity and apoptotic signaling, finally leading to cognitive deterioration.

In the current study, we compared the effects of prolonged sevoflurane exposure in wild-type mice and 3 × Tg mice; the latter are recognized as the model with pre-existing neuropathology [17]. Our results demonstrated the differential effects of sevoflurane in cognitive function, glutamate transporter, MAPK kinase signaling, and the neuronal apoptotic pathway in wild-type and 3 × Tg mice, respectively. Our data suggest the possibility that sevoflurane may have potential adverse effects on patients with pre-existing neuropathology who may require more follow-up postoperatively.

## 2. Results

### 2.1. Cognitive Function Was Impaired in 3 × Tg Mice after Sevoflurane Exposure but Not in Wild-Type Mice

**Figure 1:**
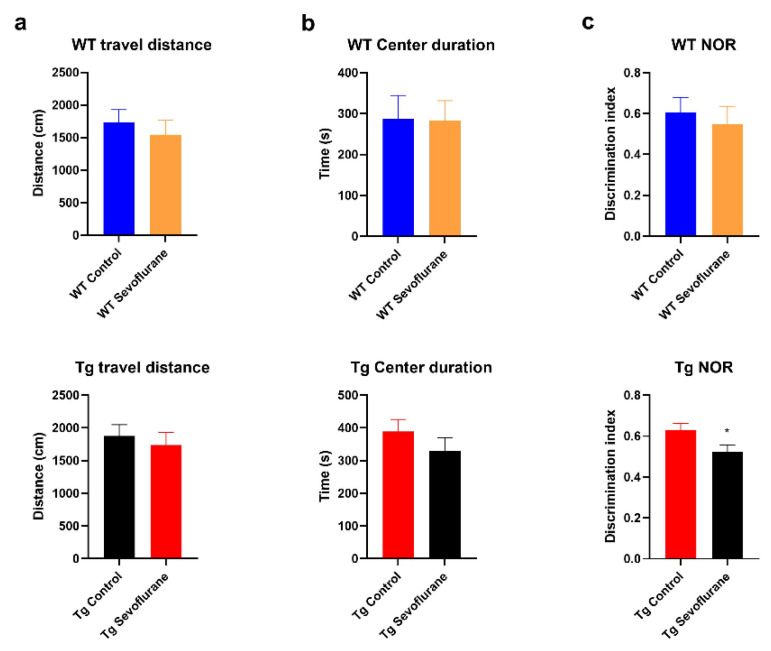
Sevoflurane induced cognitive deterioration in 3 × Tg mice but not in wild-type mice. Locomotor function, anxiety response, and cognitive function were examined using the open field test and the novel object recognition test in wild-type mice (*n* = 6) and 3 × Tg mice (*n* = 6), respectively. (**a**,**b**) No significant impact on locomotor function and anxiety was found in wild-type mice and 3 × Tg mice after sevoflurane exposure. (**c**) No significant cognitive change was observed in wild-type mice, while significant cognitive deterioration (decrease in discrimination index) was found in 3 × Tg mice after sevoflurane exposure. Data correspond to the mean ± SEM. * *p* < 0.05 in comparison to the control. Values were analyzed using the unpaired *t*-test.

The motor function and anxiety level of all mice were first examined by the open field test. No significant modulation of travel distance and central duration time was observed in both wild-type and 3 × Tg mice after sevoflurane exposure (Figure 1a,b), indicating that no locomotor deficit and anxiety was induced by sevoflurane. The cognitive function of object recognition memory was then accessed by the NOR test. In wild-type mice, no significant cognitive dysfunction was observed in the sevoflurane exposure group compared with the control group. In contrast, a significant decrease in the discrimination index was found in 3 × Tg mice after sevoflurane exposure (Figure 1c). These data first suggest that sevoflurane exposure resulted in cognitive deterioration in mice with pre-existing neuropathology background but not in the wild-type mice.

### 2.2. Differential Effects of Sevoflurane Anesthesia on Synaptic Protein Expression in Wild-Type and 3 × Tg Mice

**Figure 2:**
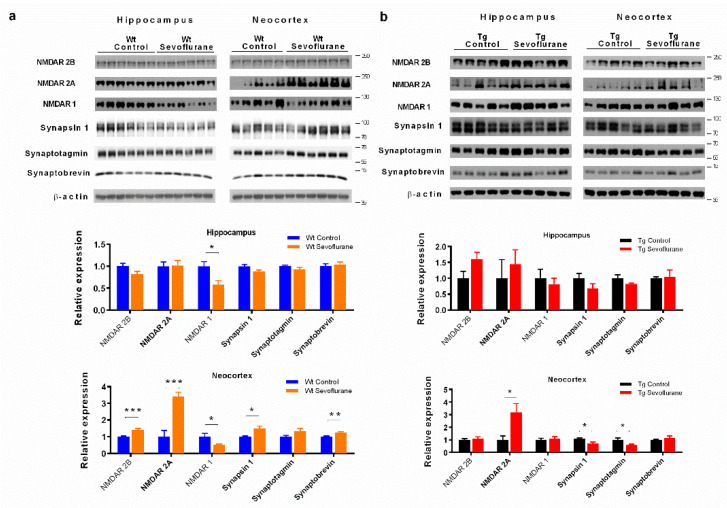
Sevoflurane altered NMDA receptors and synaptic protein expression in the hippocampus and neocortex. Synaptic NMDA receptors and vesicle proteins in hippocampus and neocortex of (**a**) wild-type mice and (**b**) 3 × Tg mice after sevoflurane exposure. Statistic graphs below show the quantification of Western blot. Data correspond to the mean ± SEM (*n* = 5–6) and represent the band densities that were normalized with the endogenous loading control. * *p* < 0.05, ** *p* < 0.01, *** *p* < 0.001 in comparison to the control. Values were analyzed using the unpaired *t*-test.

NMDA receptors and synaptic proteins in the hippocampus and neocortex were examined to investigate the effect of sevoflurane anesthesia on the glutamatergic system synaptic structure. The synaptosomal fraction from tissues was isolated and differential results were observed between the wild-type and 3 × Tg mice. In hippocampus, no significant change in the NMDA receptors and the synaptic proteins was found in both wild-type and 3 × Tg mice after sevoflurane exposure, except for a decrease in NDMA receptor 1 in the wild-type mice (Figure 2a,b). A significant increase in NMDA receptors 2A and 2B was observed in the neocortex of wild-type mice, accompanied with a decrease in NDMA receptor 1 after sevoflurane exposure (Figure 2a). Moreover, the up-regulation of vesicle proteins synapsin 1 and synaptobrevin was found (Figure 2a). Only NMDA receptor 2A, but not NMDA receptor 1 or 2B, was significantly up-regulated in the neocortex after sevoflurane exposure in 33 × Tg mice (Figure 2b). Furthermore, there was significant down-regulation of vesicle proteins synapsin 1 and synaptotagmin in the neocortex of 3 × Tg mice (Figure 2b). These data imply that sevoflurane can influence cognitive function in 3 × Tg mice through regulating NMDA receptor subunits and down-regulating synaptic proteins.

### 2.3. Sevoflurane Modulated the Expression of Vesicular Glutamate Transporters 1

**Figure 3:**
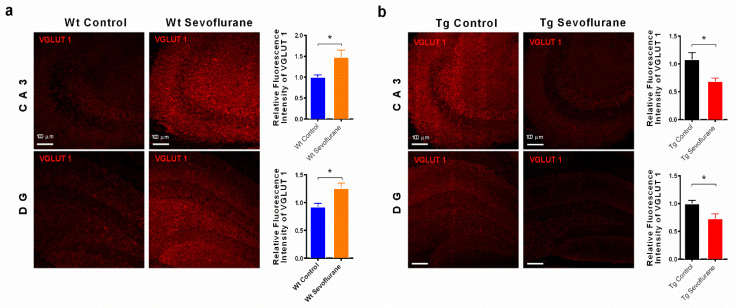
Sevoflurane reduced VGLUT1 expression in 3 × Tg mice VGLUT1 immunofluorescence in the hippocampus of (**a**) wild-type and (**b**) 3 × Tg mice. A significant reduction in VGLUT1 was found in the hippocampus of 3 × Tg mice after sevoflurane exposure, but not in wild-type mice. Bar graphs show the quantification of immunofluorescence intensity in the images. Data correspond to the mean ± SEM (*n* = 4). * *p* < 0.05 in comparison to the control. Values were analyzed using the unpaired *t*-test.

Apart from the NMDA receptor, we examined if abnormal glutamate transportation was involved in the observed cognitive impairment. Glutamate is one of the major excitatory neurotransmitters, which is the ligand for NMDA receptors [18]. Vesicular glutamate transporter 1 (VGLUT 1) is a sodium dependent phosphate transporter specially enriched in neuron-rich regions and is responsible for the recycling of glutamate from synaptic cleft [19]. A reduction in VGLUT1 results in the excess accumulation of glutamate, triggering glutamate excitotoxicity and subsequent neuronal death [14]. VGLUT1 immunofluorescence was examined in both wild-type and 3 × Tg mice after sevoflurane exposure. In the hippocampus, sevoflurane anesthesia resulted in a significantly elevated immunoreactivity of VGLUT 1 in both CA3 and DG regions in the wild-type mice (Figure 3a), but caused a significant decline in the 3 × Tg mice (Figure 3b). In the neocortex, no significant change in VGLUT1 was observed in wild-type mice (Figure 3a), while a significant reduction in VGLUT1 immunofluorescence was found in the neocortex of 3 × Tg mice (Figure 3b). These results suggest that sevoflurane can influence glutamate recycling in 3 × Tg mice and lead to cognitive dysfunction.

### 2.4. Sevoflurane Differentially Affected MAPK Signaling Pathways and Neurotrophic Factor Expression in Wild-Type and 3 × Tg Mice

Mitogen-activated protein kinases (MAPKs) are serine/threonine kinases that have been shown to control neuronal apoptosis and subsequent cognitive function [20]. Previous studies have shown that they are key players linking excessive glutamate accumulation to calcium influx and can trigger MAPK activation [21]. Thus, we examined if sevoflurane could modulate MAPK signaling activities in both wild-type and 3 × Tg mice.

**Figure 4:**
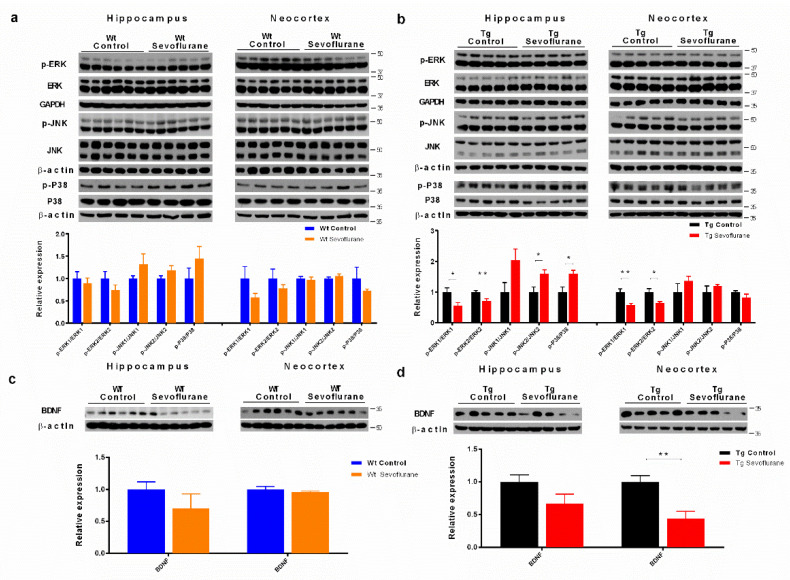
Sevoflurane differentially regulated MAPK signaling activities and BDNF in wild-type and 3 × Tg mice. Sevoflurane differentially regulated various MAP kinase phosphorylation in the hippocampus and the neocortex of (**a**) wild-type and (**b**) 3 × Tg mice. BDNF was not affected in wild-type mice (**c**) but reduced in 3 × Tg mice (**d**). Bar graphs below show the quantification of Western blot. All data correspond to the mean ± SEM (*n* = 5–6) and represent the band densities that were normalized with the endogenous loading control. * *p* < 0.05, ** *p* < 0.01 in comparison to the control. Values were analyzed using the unpaired *t*-test.

In wild-type mice, no significant changes in phosphorylated ERK, JNK, and p38 were found in either the hippocampus or neocortex after sevoflurane exposure (Figure 4a). On the other hand, the significant down-regulation of phosphorylated ERK and up-regulation of JNK was observed in both hippocampus and in the neocortex of 3 × Tg mice (Figure 4b). To further our observations, brain-derived neurotrophic factor (BDNF) was examined, as it is one of the ERK pathway downstream molecules and is a well-known neurotrophic factor that facilitates neuronal survival and development [22]. In wild-type mice, no significant change in BDNF was found (Figure 4c). In 3 × Tg mice, however, the down-regulation of BDNF was observed in the neocortex (Figure 4d). These results show that sevoflurane may induce cognitive deterioration in 3 × Tg mice through regulating MAPK pathways and reducing BDNF expression.

### 2.5. Sevoflurane Exerted Different Effects on Apoptosis in Hippocampi and Neocortices of Wild-Type and 3 × Tg Mice

**Figure 5:**
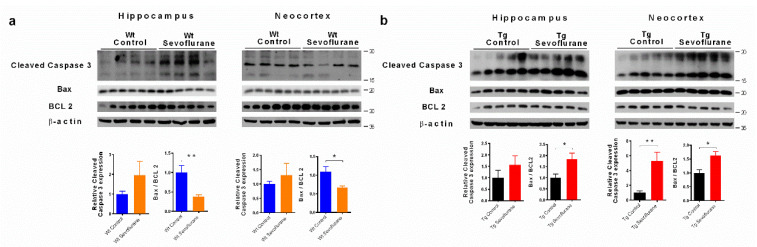
Sevoflurane activated the apoptotic pathway in 3 × Tg but not in wild-type mice. Cleaved caspase 3 and BAX/Bcl2 proteins were examined in the hippocampus and neocortex of wild-type and 3 × Tg mice. (**a**) No significant change in the cleaved caspase 3 was in line with a reduction in the BAX/Bcl2 ratio in wild-type mice. (**b**) Significant elevation of cleaved caspase 3 protein and BAX/Bcl2 ratio in 3 × Tg mice. Bar graphs below show the quantification of Western blot. Data correspond to the mean ± SEM (*n* = 5–6) and represent the band densities that were normalized with the endogenous loading control. * *p* < 0.05, ** *p* < 0.01 in comparison to the control. Values were analyzed using the unpaired *t*-test.

**Figure 6:**
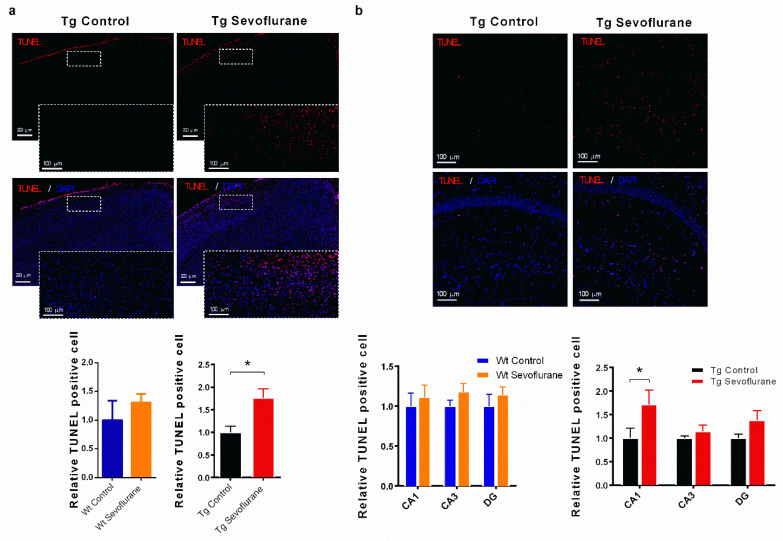
DNA damage in 3 × Tg mice brain after sevoflurane exposure but not in wild-type mice. TUNEL staining indicated the DNA fragmentation in the (**a**) neocortex and (**b**) hippocampus of 3 × Tg mice. A significant increase in TUNEL-positive cells was found in 3 × Tg mice but not in wild-type mice (**a**,**b**). Statistic graphs show the quantification of immunofluorescence intensities in the images. Data correspond to the mean ± SEM (*n* = 4). * *p* < 0.05 in comparison to the control. Values were analyzed using the unpaired *t*-test.

As MAPK activities could be linked to apoptosis, we examined if sevoflurane triggered apoptosis in our animal models [23,24]. In wild-type mice, although no significant modulation of caspase 3 cleavage was found, there was a significant reduction in the Bax/Bcl2 ratio in both the hippocampus and neocortex after sevoflurane exposure (Figure 5a), suggesting that apoptosis may be inhibited by sevoflurane in wild-type mice. In contrast, significant up-regulations of cleaved caspase 3 (in neocortex) and Bax/Bcl2 ratio (in both hippocampus and neocortex) were found in 3 × Tg mice after sevoflurane exposure (Figure 5b), indicating that sevoflurane induced apoptosis in 3 × Tg mice. We further confirmed apoptotic cell death in 3 × Tg mice using the TUNEL assay. Our results demonstrate that there was significant increase in TUNEL-positive cells in both neocortex and hippocampus compared with the sham group (Figure 6a,b), which supported the pro-apoptotic effect of sevoflurane in 3 × Tg mice.

## 3. Discussion

Conflicting results have been reported regarding the effects of volatile anesthesia on postoperative cognition, arguably due to a lack of consideration for variation in individual susceptibility to potentially adverse effects of sevoflurane based on differences in pathological background. This may in part account for mild cognitive impairment and advance aged being known risk factors for PND [10,25]. To further investigate this issue experimentally, wild-type and 3 × Tg mice, respectively, representing subjects with normal and likely early neuropathological changes, were used. Three-month-old 3 × Tg mice were chosen as early neuropathology, including Aβ aggregation and tau hyperphosphorylation, have been reported in the brain [17]. Our data support that sevoflurane had no significant impact on wild-type mice, while distinct adverse effects were observed in the brains of 3 × Tg mice. With pre-existing neuropathology, sevoflurane exposure was shown to modulate the expression of different NMDA receptor subunits, vesicular glutamate transporter, and synaptic vesicle proteins in the CNS. The agent also activated JNK and inhibited ERK pathways, which further induced neuronal apoptosis. Our findings identify a particular risk factor that pre-existing neuropathology may increase the susceptibility of sevoflurane-induced cognitive deterioration in an experimental animal model. This also explains the previous findings that aging is a risk factor for PND, as different neuropathological hallmarks including tau aggregation, deposition of amyloid plaque, and neurofibrillary tangles are usually found with increasing age.

The synapse is considered as the primary site of neurodegeneration prior to cognitive deficit symptoms [26]. Cognitive deficit is correlated with manifestations of synaptic dysfunction, such as structural changes, neurotransmission dysfunction, and a reduction in synaptic strength. In Alzheimer’s disease (AD), levels of functional synaptic proteins correlate significantly with the severity of memory loss [27], and AD begins with subtle alterations of hippocampal synaptic efficacy prior to frank neuronal degeneration [28,29]. The regional loss of synapses, particularly within the neocortex and hippocampus, is characterized in AD and it is strongly correlated with the progress of cognitive impairment [30]. From our results, no significant impact on cognitive function was observed in wild-type mice after sevoflurane exposure. On the contrary, sevoflurane exposure resulted in the cognitive deterioration in 3 × Tg mice. In view of this finding, we first examined the synaptic protein expressions, including synaptic vesicle proteins, NMDA receptors, and vesicular glutamate transporters.

We demonstrated the downregulation of synapsin 1 and synaptotagmin in 3 × Tg mice after sevoflurane exposure (Figure 2), which indicates that a disruption in synaptic transmission may contribute to the observed cognitive dysfunction. Synapsin 1 is a synaptic vesicle-associated protein that plays an important role in axonogenesis and synaptogenesis [31]. On the other hand, synaptotagmin is a membrane-bound synaptic protein involved in the calcium sensing machinery and vesicle fusion [32]. These synaptic proteins are crucial in dendritic spine formation and the plasticity of learning [33,34]. Our results demonstrate a significant decrease in synapsin 1 and synaptotagmin in 3 × Tg mice, but not in wild-type mice after sevoflurane exposure. These data imply that sevoflurane interrupted the synaptic function in subjects with pre-existing neuropathology.

We also showed that NMDA receptor 1 was reduced in both wild-type and 3 × Tg mice after sevoflurane exposure (Figure 2). However, an increase in NMDA receptor 2A and 2B was observed in wild-type mice, while only NMDA receptor 2A was increased in 3 × Tg mice (Figure 2). The functional NMDA receptor is an ionotropic glutamate receptor composed of both NMDA receptor 1 and NMDA receptor 2 subunits [35]. Changes in the composition of NMDA receptors on synaptic membrane could influence synaptic strength and even synaptic degeneration. This observation is consistent with the previous findings which indicate that an increase in 2A subunits can result in the increased vulnerability of neurons to glutamate excitotoxicity [36]. Moreover, long-term memory deficits were found in mice overexpressing the 2A unit by increasing the 2A/2B ratio and compressing long-term depression [37]. Our data, together with previous findings, suggest that sevoflurane may lead to an imbalance in the 2A and 2B ratio in 3 × Tg mice and have implications for sevoflurane-induced cognitive deficit in 3 × Tg mice.

Apart from the modulation of NMDA receptors, a significant reduction in vesicular glutamate transporter 1 (VGLUT1) was observed in the brain of 3 × Tg mice, while there was no significant change in VGLUT1 in wild-type mice after sevoflurane exposure (Figure 3). VGLUT1 is an important membrane transporter protein responsible for the uptake and release of glutamate [38]. The dysfunction of VGLUT leads to the over-accumulation of glutamate and the disruption of glutamate homeostasis, which results in the over-activation of NMDA receptors. These events finally result in triggering glutamate excitotoxicity with excess intracellular calcium influx and subsequent signaling modulation [39]. In the postmortem brain of AD patients, a loss of VGLUT1 was found [40]. These observations agree with our current results of down-regulation of VGLUT1 in 3 × Tg mice brain (Figure 3). In line with our previous findings of NMDA receptor regulation, it is possible that sevoflurane may have significant impact on the glutamatergic system and cause excitotoxicity in the brains of 3 × Tg mice. Apart from glutamate transporter, the removal of glutamate from synaptic cleft can be achieved after decarboxylating glutamate into gamma-aminobutyric acid (GABA) through the glutamate decarboxylase (GAD) [41]. GAD exists in two isoforms with molecular weights of GAD65 and GAD67. Although the GAD expression has not been examined in current study, sevoflurane was shown to have minimal impact on the expression of GAD67 in a previous report [42]. Those findings suggest that sevoflurane may influence the glutamatergic system through its transporter, but not by its catalytic conversion.

Following glutamate excitotoxicity, intracellular calcium influx occurred through NMDA receptors. These accumulated calcium ions could lead to the activation of calcium-dependent kinases, such as Ca2+/calmodulin-dependent protein kinase II (CamKII), which subsequently regulate different MAPK, such as JNK [15] and ERK [43]. We further investigated if sevoflurane modulated MAPK signaling in 3 × Tg mice. From Western blotting results, a significant up-regulation of phosphorylated JNK and down-regulation of phosphorylated ERK was found in the 3 × Tg mice after sevoflurane exposure, but not in wild-type mice (Figure 4). These findings are in agreement with a previous report which stipulates that sevoflurane induced cognitive dysfunction through MAPK pathway modulation [44]. ERK and JNK/p38 have been considered to have opposing effects in terms of regulating neuronal apoptosis and survival [45]. ERK is a widely conserved serine/threonine MAP kinase which can be activated by growth factors and is involved in cell differentiation, cell proliferation, and cell survival [46]. The phosphorylation of ERK on threonine and tyrosine residues results in an increase in the transcriptional activities by phosphorylating transcriptional factors, therefore increasing the synthesis of neurotrophic factors, including BDNF [22], and exerting anti-apoptotic effects [45]. Consistent with those observations, we observed a significant reduction in phosphorylated ERK and BDNF in the brains of 3 × Tg mice, but not of wild-type mice (Figure 4), implying that sevoflurane may affect cognitive function not only through synaptic deficit, but also through inhibiting neurotrophic factors synthesis. On the other hand, JNK is another MAP kinase which mediates neuroinflammation [47] and apoptotic program [45]. Activation of JNK has been shown to increase the expression of pro-inflammatory cytokines such as TNF-α and IL-1β, which could inhibit both synaptic and neuronal function [48]. Moreover, JNK activation can result in the phosphorylation of p38 kinase and induce apoptosis in the brain [45]. We observed up-regulation of JNK and p38 phosphorylation in the brain of 3 × Tg mice after sevoflurane exposure (Figure 4). These data suggest that sevoflurane may exacerbate neuronal dysfunction in 3 × Tg mice through the activation of JNK/p38 and the inhibition of ERK pathways, demonstrating the deleterious effects of sevoflurane in subjects with pre-existing neuropathological damage.

Both JNK activation and ERK inhibition could lead to neuronal apoptosis [45], which contributes to neuronal cell death and cognitive dysfunction. No significant expression of cleaved caspase 3 was seen in wild-type mice, but significant up-regulation was observed in the neocortex of 3 × Tg mice (Figure 5). However, up-regulation of the BAX/Bcl2 ratio was observed in the 3 × Tg mice after sevoflurane exposure, whereas no significant change was observed in wild-type mice (Figure 5). BAX is a key component for cellular apoptosis which can be activated by JNK [49]. When stimulated, it will be translocated to the mitochondrial membrane and increase its permeability, which could trigger apoptosis [50]. In contrast, anti-apoptotic Bcl2 prevents the leakage of cytochrome c from mitochondria, thus inhibiting apoptosis and facilitating cell survival [51]. In addition, more TUNEL-positive cells were found in 3 × Tg mice with sevoflurane exposure (Figure 6). Our findings further demonstrate that sevoflurane may exacerbate cognitive dysfunction in 3 × Tg mice through inducing apoptosis, together with the synaptic dysfunction in the central nervous system. As the neurotoxicity of sevoflurane was observed in our animal model, alternatives of general anesthesia can be considered when it is applied in patients with similar conditions. Propofol is another widely used general anesthesia that can induce GABAergic activities in the CNS [52]. Previous reports showed that minimal impact on behavior and pathological changes were observed in Alzheimer’s disease mice with propofol treatment [53,54]. These findings, together with our data, suggest that propofol may be used as an alternative of sevoflurane for patients with pre-existing pathological background.

## 4. Materials and Methods

### 4.1. Animals

Three-month-old male 3 × Tg mice (triple transgenic B6; 129-Psen1tm1Mpm Tg (APPSwe, tauP301L) 1L fa/J) and wild-type C57 mice were obtained from the Center of Comparative Medicine Research in The University of Hong Kong. All animals were housed in a temperature-controlled room at 20–22 °C, with humidity of 50 ± 10%, and were kept on a 12–12 h light–dark cycle. All animals had access to food and water ad libitum, and they underwent an acclimatization period for one week before being employed in the experiment. All experimental protocols were approved by the Department of Health, HKSAR, and the Faculty Committee on the Use of Live Animals in Teaching and Research (CULATR) at The University of Hong Kong. All animal houses and facilities in The University of Hong Kong were accredited by the AAALAC International, and its regulations were followed to ensure that all animals experience minimal impact from environmental stress. All efforts were made to minimize animal suffering.

### 4.2. Sevoflurane Exposure

Mice were placed on a warming mat in an acrylic glass chamber filled with sevoflurane (Abbvie, North Chicago, IN USA), with the agent delivered in oxygen using rodent inhalation anesthesia apparatus (Harvard Apparatus, Holliston, MA, USA), equipped with a vaporizer and a purpose-made gas delivery and scavenging system. After 5 min induction of anesthesia in the Perspex induction chamber (5% sevoflurane, 800 mL/min airflow), anesthesia was maintained for 2 h via a nose mask (2.5% sevoflurane, 800 mL/min airflow) with the mice breathing spontaneously and with rectal temperature kept between 37 and 38 °C. Respiration and heartbeat rates were monitored throughout the exposure. Mice were then allowed to recover and transferred back to their home cages until further behavioral experiments.

### 4.3. Open Field Test

The open field test was used to assess locomotor activities and the presence of anxiety in rodents [55,56]. On day 5 post-exposure with sevoflurane, mice were placed in the center of a plastic open box (40 cm wide, 40 cm long, 40 cm high), and were allowed to freely explore. The length of travel distance and the time of center duration were captured using a video recording system. The mice behavior was then analyzed using the Panlab SMART VIDEO TRACKING Software (Harvard Apparatus, Holliston, MA, USA). In the video, the base of the box was divided into nine equal grids (13.3 cm × 13.3 cm). The locomotor function was accessed by measuring the total travel distance in the box within 10 min. The time of center duration (stay within center grid) was recorded which indicated a less anxious state of the mice.

### 4.4. Novel Object Recognition Test

Object recognition memory was determined using a novel object recognition test (NOR), as described with modifications [57]. Briefly, on day 6 post-exposure, mice were put in the open box, similar to in the open field test. Two identical objects were placed in the box and mice were allowed to explore and familiarize both objects for 10 min. Twenty-four hours after familiarization, one of the two objects was replaced with a novel object. The mice were allowed to explore both objects for 10 min. The behavior of the mice was captured using a video recording system and analyzed using the software. The discrimination index was calculated by the formula: Tn/Tt, where Tn is the time of exploring novel object, while Tt is the total time of exploring novel and familiarized objects. A higher discrimination index indicated a better recognition memory of the mice. For both open field and novel object recognition tests, analyzers were blinded from the grouping of animals when they performed analysis on the video recording. Exclusion criteria for NOR included the inactive subject exploration of animals (total subject exploration time <20 s) and the recognition bias on a specific side of subject in the familiarization phase.

### 4.5. Preparation of the Whole Tissue Lysate and Synaptosomal Fraction

After the NOR test, mice were sacrificed using CO_2_ asphyxiation in accordance with the guidelines of the American Veterinary Medical Association. Hippocampal and neocortical tissues were dissected from the left hemispheres. Whole protein extracts were prepared by mechanically homogenizing the tissues in RIPA buffer containing protease and phosphatase inhibitors (Roche, Indianapolis, IN, USA). The synaptosomal fraction was freshly prepared using the Syn-PERTM Synaptic Protein Extraction Reagent (Thermofisher Scientific, Waltham, MA, USA) plus protease and phosphatase inhibitors (Roche, Indianapolis, IN, USA) according to the manufacturer’s instructions.

### 4.6. SDS-PAGE and Western-Blot Analysis

SDS-PAGE was performed as previously described [58]. Both whole brain lysates and synaptosomal fraction were subjected to electrophoresis and transferred onto PVDF membranes, respectively. Non-specific binding sites were blocked with 5% non-fat dry milk for 1 h, followed by incubation with primary antibodies overnight at 4 °C. After 2 h of incubation with horseradish peroxidase-conjugated secondary antibodies (DAKO, Glostrup, Denmark), the band signal intensity was subsequently visualized using the chemiluminescence substrate (Advansta, San Jose, CA, USA). All immunoblots were normalized for gel loading with β-actin, GAPDH, or α-tubulin antibodies. The intensities of chemiluminescent bands were measured using Image-J software (National Institutes of Health, Bethesda, MD, USA). The levels of different kinases were presented as the ratio of phosphokinase to total kinase, after being normalized by the internal control.

### 4.7. Immunofluorescence Staining

After CO2 asphyxiation, mice were perfused with cold 0.9% saline and the right hemispheres were immediately fixed with cold 4% paraformaldehyde in phosphate buffer (PB: 0.1 M, pH 7.4). Brain tissues were fixed in 4% paraformaldehyde for 72 h, and 20 µm-thick frozen sections were made. Sections were treated with 0.01 M citrate buffer (pH 6.0) with 0.1% Tween 20 at 90 °C for 15 min for antigen retrieval. After blocking with 10% normal goat serum for 2 h at room temperature, sections were incubated with primary antibody (VGLUT1, 1:200; Merck, Kenilworth, NJ, USA) at 4 °C overnight. Sections were then incubated with secondary antibody conjugated with Alexa 568 (1:400; Thermofisher Scientific, Waltham, MA, USA) for 2 h at room temperature. Slides were observed under a laser-scanning confocal fluorescent microscope (Carl Zeiss LSM 880, Oberkochen, Germany), equipped with ZEN light software. Z-stack images were acquired and analyzed using Image-J software (Version 1.53i, U. S. National Institutes of Health, Bethesda, MD, USA). All quantitative analyses were performed on at least four images acquired from at least four serial sections per animal from at least two independent experiments.

### 4.8. TUNEL Assay

Apoptosis was detected using the TdT-mediated dUTP nick-end labeling (TUNEL) assay (Roche, Indianapolis, IN, USA), which labels the cut ends of DNA fragments in the nuclei of apoptotic cells. The counterstaining was performed with 5 μM DAPI (Sigma-Aldrich, St. Louis, MO, USA). Quantification of apoptosis in terms of TUNEL intensities and morphology was performed in 3–5 hippocampal and frontal cortical sections.

### 4.9. Statistical Analysis

Normality of the data and homogeneity of group variances were assessed using the D’Agostino–Pearson omnibus normality test, the Shapiro–Wilk normality test, and the Kolmogorov–Smirnov test, respectively. After assuming that normality was not met, the non-parametric test was employed. Data were analyzed using an unpaired two-tailed t test, with the statistic software GraphPad Prism (Graph Pad Software Inc., La Jolla, CA, USA). Data were presented as the mean ± standard error of the mean (SEM). Statistical significance was determined when *p* ≤ 0.05.

## 5. Conclusions

In conclusion, sevoflurane exacerbated cognitive dysfunction in the mice with pre-existing neuropathology, but with minimal impact on wild-type mice. This dysfunction was accompanied by the modulation of NMDA receptors, synaptic vesicles proteins, MAPK signaling, and subsequent neuronal apoptosis. Our results add to in vivo evidence supporting the differential impact of sevoflurane on cognition, which depends on the pre-existing neuropathology background. Since other risk factors such as metabolic disorder also gain more focus in anesthesia-induced cognitive decline, our findings together can alert physicians to focus on the preoperative assessment and choice of drugs. In addition, our results indicate the potential involvement of glutamate homeostasis in the current model. Further therapeutic strategies with potential translational value, such as enhancing glutamate transportation or blocking glutamate excitotoxicity, could also be examined.

## Data Availability

The data presented in this study are available on request from the corresponding author.
